# Bee Venom and Its Sub-Components: Characterization, Pharmacology, and Therapeutics

**DOI:** 10.3390/toxins13030191

**Published:** 2021-03-07

**Authors:** Woojin Kim

**Affiliations:** Department of Physiology, College of Korean Medicine, Kyung Hee University, Seoul 02453, Korea; wjkim@khu.ac.kr

Bee venom, which is a complex substance produced by *Apis mellifera*, is widely used to treat various diseases, such as pain [1], inflammation [2], and cancer [3]. In addition, along with bee venom, a variety of sub-components are being used as therapeutic agents. However, their safety is still an important concern [4], and their pharmaceutical characterization and mechanism of action are not clearly understood. Therefore, further investigation is required. Bee venom contains various types of peptides (i.e., melittin, apamin), enzymes (i.e., phospholipase A_2_ (PLA_2_), hyaluronidase), biologically active amines (i.e., histamine and epinephrine), and non-peptides (i.e., amino acids). Among them, melittin is a major compound, as it consists of 40–50% of the dry weight of bee venom. PLA_2_ and apamin are other major components, accounting for 10–12% and 2–3% of dry bee venom, respectively [5].

Thus, this Special Issue has focused on the pharmaceutical characterization and therapeutic effects of bee venom and its sub-components. A total of 11 studies were published. Three reviews provided a good overview of the therapeutic effect of bee venom [6,7,8], and eight original research articles focused on bee venom, melittin, PLA_2_, and apamin [9,10,11,12,13,14,15,16].

Among these eight papers, four dealt with melittin. First, using an ultra-performance liquid chromatography-quadrupole time-of-flight mass spectrometry (UPLC-QqTOF-MS) method, Huang et al. [11] reported that the melittin content in Asian honeybee venom samples collected from two different zones of China (i.e., Wuhan and Jilin) makes up 33.9–46.23% of their dry weight. They suggested that seasonal and environmental factors should be considered to obtain the highest melittin content from the bee venom. Second, to maximize the anticancer effect of melittin, Cheng and Xu [10] demonstrated a method to deliver melittin more efficiently and stably by developing a redox-sensitive polymer-based nanocomplex. They reported that polymer/melittin nanocomplexes showed increased cytotoxicity compared to free melittin. Third, Zorila et al. [16] used spectral analysis of Laurdan fluorescence to demonstrate that melittin could induce local order changes in artificial and biological membranes. Finally, Kim et al. [12] examined the role of melittin in cisplatin-induced acute kidney injury and showed that intraperitoneal administration of different doses of melittin could inhibit cisplatin-induced increase in creatinine and blood urea nitrogen by regulating M2 macrophage expression.

Two studies assessed the therapeutic effects of PLA_2_ and apamin. The role of PLA_2_ in the apoptotic signaling pathway in regulatory T cell (Treg) populations was examined by Baek et al. [9]. They demonstrated that PLA_2_ treatment can upregulate the expression of anti-apoptotic molecules, such as cytotoxic T-lymphocyte antigen 4 and programmed death 1. Moreover, the survival rate of Tregs increased following PLA_2_ administration. Apamin was investigated by Gu et al. [6]. They explored the therapeutic effect of apamin by reviewing nine published papers that analyzed its effect on apoptosis, fibrosis, and central nervous system dysregulation.

Three other studies focused on the effects of bee venom acupuncture (BVA). Lin and Hsieh [8] analyzed the anti-inflammatory, anti-apoptotic, and analgesic effects of bee venom injected at various acupoints. A study by Li et al. [14] examined whether combined treatment with BVA and venlafaxine, a well-known serotonin and norepinephrine re-uptake inhibitor, could produce synergetic effects. They reported that the combined therapy resulted in long-lasting and additive anti-allodynic effects. In their study, cold and mechanical allodynia were induced by intraperitoneal injection of paclitaxel in mice. Furthermore, Lee et al. [13] demonstrated that BVA could alleviate acute cold and mechanical allodynia induced by oxaliplatin administration by increasing the lowered action potential threshold in A-fiber but not in C-fiber dorsal root ganglia neuronal cells. Li et al. and Lee et al. suggested that bee venom could be used against chemotherapy-induced neuropathic pain, as both paclitaxel and oxaliplatin are widely used chemotherapeutic agents.

Finally, two studies focused on the whole honeybee venom. Pawlek et al. [15] demonstrated that citric acid was the most abundant acid, whereas glutaric acid and kynurenic acid were the lowest organic acids present in the honeybee venom. A review study by El-Seedi et al. [7] focused on the antimicrobial properties of honeybee venom. They reviewed all studies conducted in vivo and in vitro against bacteria, viruses, and fungi.
